# Red algae acclimate to low light by modifying phycobilisome composition to maintain efficient light harvesting

**DOI:** 10.1186/s12915-022-01480-3

**Published:** 2022-12-27

**Authors:** Sofie E. Voerman, Arvydas Ruseckas, Graham A. Turnbull, Ifor D. W. Samuel, Heidi L. Burdett

**Affiliations:** 1 Lyell Centre for Earth and Marine Science and Technology, Edinburgh, EH14 4BA UK; 2 School of Energy, Geoscience, Infrastructure and Society, Heriot-Watt University, Edinburgh, EH14 4AS UK; 3 Organic Semiconductor Centre, SUPA, School of Physics and Astronomy, University of St Andrews, St Andrews, KY16 9SS UK; 4 Present Address: Department of Ecology and Environmental Sciences, Umeå University, Umeå, Sweden; 5 Umeå Marine Sciences Centre, Umeå University, Norrbyn, Sweden

**Keywords:** Coralline Algae, Photosynthesis, Phycobilisome, Mesophotic, Fluorescence, Photosystem, Photo-acclimation, Chromo-acclimation, Maerl, Rhodolith

## Abstract

**Background:**

Despite a global prevalence of photosynthetic organisms in the ocean’s mesophotic zone (30–200+ m depth), the mechanisms that enable photosynthesis to proceed in this low light environment are poorly defined. Red coralline algae are the deepest known marine benthic macroalgae — here we investigated the light harvesting mechanism and mesophotic acclimatory response of the red coralline alga *Lithothamnion glaciale*.

**Results:**

Following initial absorption by phycourobilin and phycoerythrobilin in phycoerythrin, energy was transferred from the phycobilisome to photosystems I and II within 120 ps. This enabled delivery of 94% of excitations to reaction centres. Low light intensity, and to a lesser extent a mesophotic spectrum, caused significant acclimatory change in chromophores and biliproteins, including a 10% increase in phycoerythrin light harvesting capacity and a 20% reduction in chlorophyll-*a* concentration and photon requirements for photosystems I and II. The rate of energy transfer remained consistent across experimental treatments, indicating an acclimatory response that maintains energy transfer.

**Conclusions:**

Our results demonstrate that responsive light harvesting by phycobilisomes and photosystem functional acclimation are key to red algal success in the mesophotic zone.

**Supplementary information:**

The online version contains supplementary material available at 10.1186/s12915-022-01480-3.

## Background

The mesophotic zone − arbitrarily defined as > 30 m water depth and/or < 1% surface light intensity to the lower limit of light penetration in the oceans [1] − is a critical ‘transition’ zone between the ocean surface and the deep sea. Recent developments in ocean exploration have begun to reveal the extent of mesophotic benthic photosynthetic organisms (such as corals and algae) [2]. Despite low light levels, these organisms create complex habitats of significant ecological importance, supporting high biodiversity [3–5] and efficient biogeochemical cycling [6–8].

Light attenuation in the oceans with depth is characterised by both a decline in the intensity of light available for photosynthesis (photosynthetic active radiation [PAR], 400–700 nm) and a narrowing of the spectral composition — resulting in a blue dominance in oligotrophic oceanic waters [9, 10]. Mesophotic photosynthetic organisms therefore have to contend with both reduced light intensity and bandwidth compared to their shallow-water counterparts [11, 12] to meet their energy requirements. Although organismal energy requirements may be reduced in the mesophotic (e.g. lower growth rates), understanding the bottom-up mechanisms that drive an organism’s chemical energy supply is a first and crucial step in understanding its presence at extreme depth. Light availability is therefore the primary limiting factor in the maximum depth distribution of macroalgae [13], which in turn negatively impacts the ecological and biogeochemical benefits they can provide [5]. To overcome the limitations of reduced irradiance, algae are known to increase their photosynthetic efficiency and reduce their minimum light requirements (e.g. [14–17]).

Tolerance to a mesophotic light regime therefore poses a significant competitive advantage for increasing an organism’s distributional extent, but the physiological mechanism for how this tolerance is achieved remains unclear. Red coralline algae are the deepest known habitat-forming algae, found even at 270 + m depth and at irradiance levels less than 0.001% of those found at the water surface [13, 17–19]. As with other red algae and cyanobacteria, red coralline algae harbour multi-pigment light harvesting complexes in addition to chlorophyll-*a* [20]. Phycobilisomes consist of phycoerythrin (PE), phycocyanin (PC), allophycocyanin (APC), and linker proteins [21], precisely arranged in an energy absorption cascade towards subsequent energy transfer to chlorophyll-*a* in photosystems I and II (PSI and PSII) [21, 22]. These extra light harvesting molecules effectively increase the bandwidth of photosynthetically available light energy, particularly in the mid-range of the visible light spectrum [15, 23] — which becomes increasingly dominant at increasing water depth [9]. The mesophotic success of red coralline algae has been attributed to their low growth rates [24] coupled with the light harvesting advantages posed by phycobilisomes [25]. However, the effective light harvesting mechanism adopted by these algae remains undefined [2, 17, 26] and it is not known how phycobilisome energy transfer cascades respond to environmental change [27], limiting our ability to explain the mesophotic success of red coralline algae.

Here, we used time-resolved fluorescence and its excitation spectroscopies to identify for the first time the excitation energy transfer (EET) pathway through the phycobilisomes of red coralline algae (*Lithothamnion glaciale*). Then, we investigated how this pathway mechanistically responds to environmentally-relevant regimes of light intensity and spectral composition that were representative of mesophotic and shallow water conditions. This provided new insight into mesophotic acclimation of red algal photosynthesis and phycobilisome function. As the world’s deepest known habitat-forming algae, this provides a physiological explanation for the maintenance of red coralline algal photosynthesis in extreme low light ocean environments.

## Results

### Characterisation of the *L. glaciale* light harvesting mechanism

**Fig. 1:**
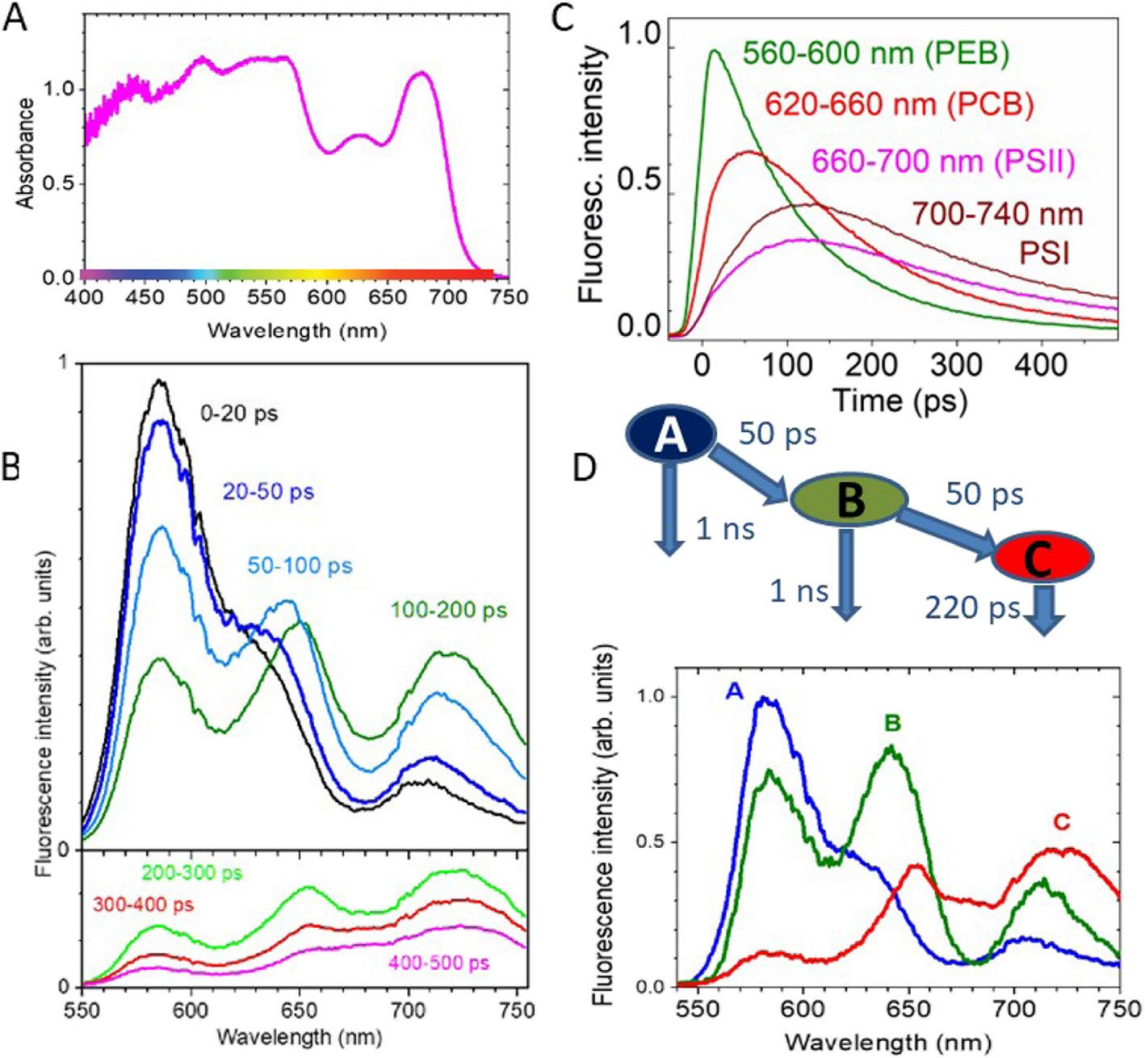
Phycobilisome excitation energy transfer in *L. glaciale*. **A** A typical absorbance spectrum from *L. glaciale*, obtained from measured reflectivity. **B** Representative time-resolved fluorescence spectra of *L. glaciale* after excitation with 200 fs light pulses at 515 nm across time windows up to 500 ps. **C** Fluorescence kinetics of chromophore phycoerythrobilin (PEB), phycocyanobilin (PCB) and photosysystems II and I. **D** The compartmental model of consecutive energy transfer (A → B → C) applied to fit the time-resolved fluorescence spectra with time constants and spectral amplitudes of each compartment obtained from fitting. The down-pointing arrows from A and B compartments represent the natural decay of excitons in phycoerythrin (PE) and phycocyanin (PC) hexamers with a time constant of 1 ns which is taken from the fluorescence decay of PE in water (cf. Additional File 1: Figure S2), whilst the 220 ps decay time of the C compartment is obtained from fitting and represents charge separation in photosystems

We first established the baseline light harvesting pathway of the temperate red coralline alga *L. glaciale* in terms of energy transfer through the phycobilisome. Strong absorption of visible light up to 700 nm was observed (Fig. 1A). Although the absorption spectra of known red algal pigments overlap, absorption maxima at 440 and 680 nm are observed, attributed to the Soret and Q_y_ chlorophyll-*a* bands, respectively. Absorption peaks at ~ 565 and 630 nm are characteristic of the phycobiliproteins PE and PC, respectively. Additional PE absorption is also observed at 495 nm (attributed primarily to PE containing phycourobilin [PUB], and perhaps some carotenoid signal) and 535 nm.

The mechanism of EET to PSI and PSII was determined using time-resolved fluorescence spectroscopy with excitation at 515 nm where absorption is dominated by PE (Fig. 1B). At 0–20 ps, the spectrum is dominated by a strong fluorescence band between 550 and 610 nm — attributed to PE (confirmed via comparison to PE dissolved in water; Additional File 1: Figure S1). At 50–200 ps after excitation, the intensity of the phycoerythrobilin (PEB) band decreased and two other fluorescence bands intensified: (1) between 620 and 660 nm — attributed to phycocyanobilin (PCB) and (2) 660–740 nm, attributed to chlorophyll-*a* fluorescence in PSII and PSI (Fig. 1C). An intensity minimum is apparent at around 680 nm, just between the fluorescence bands of PC and chlorophyll-*a*, where APC subunits ApcD and ApcE/ApcF (essential for energy transfer to PSI and PSII [28]) are known to emit. This indicates that transient fluorescence from these terminal emitters is too short lived to observe and implies very fast energy transfer from APC to chlorophyll-*a* in photosystems. Our measurements did not separate PSII and PSI emission spectrally, showing just a single broad fluorescence band between 680 and 760 nm. We attributed this band to both PSII and PSI based on the following consideration: The maximum of this band red-shifts from ~ 710 nm at 20–50 ps after excitation to ~ 720 nm at 100–200 ps, explained by energy transfer to lower energy sites within PSI. Yet, even at long timescales (200–500 ps), we still see fluorescence in the 680–700 nm region. This is too blue to originate from PSI because by that time excitons are expected to have reached the lowest energy sites within PSI and therefore show similar spectra to the steady state fluorescence of PSI (> 700 nm) [29]. We therefore attribute the short wavelength section of the 680–700-nm band to PSII. The simultaneous appearance of PSI and PSII fluorescence in the 50–100 ps spectrum indicates that energy was transferred to both photosystems at a similar rate. Fluorescence bands of PC and chlorophyll-*a* show a red-shift over time indicating energy transfer to lower energy sites within each complex. At longer timescales (200–500 ps) all fluorescence features reduce in strength (Fig. 1B, C), attributed to both slower energy transfer from PE chromophores distal to the phycobilisome core and charge separation in the photosystems.

A compartmental model of consecutive energy transfer (A → B → C) was used to create a global analysis of energy transfer through the photo-apparatus (Fig. 1D). The natural decay times of A and B compartments were fixed to 1 ns — representative of the measured exciton lifetime of PE dissolved in water (Additional File 1:Figure S2). Good fits were obtained with a time constant of 50 ps for A → B and B → C energy transfer. The A compartment is dominated by PE emission at 560–610 nm. The B compartment showed lower intensity of PE emission compared to the A compartment by ~ 30% and stronger emission of PC at 620–660 nm and chlorophyll-*a* in PSI and PSII (680–760 nm). This implies that ~ 30% of the energy is transferred from PE to PC and to photosystems on a time scale of 50 ps. The C compartment shows a weak PE emission, a PC band with its peak red-shifted to 655 nm, a shoulder at 660–690 nm, which we attribute to APC, and a strong chlorophyll-*a* emission (680–760 nm). The modelled spectra indicate that the primary energy transfer pathways are PE → PC → APC → PSI&PSII and PE → APC → PSI&PSII (Fig. 1D). The 220 ps decay time of the C compartment represents charge separation in the photosystems. The decay time of PE fluorescence in *L. glaciale* from 560 to 610 nm indicates an energy transfer time of ~ 120 ps from PE to the APC core and to the photosystems (Additional File 1: Figure S2). This is more than ten times faster than the exciton decay time of the PE complex dissolved in water (1500 ps). Light harvesting efficiency by the phycobilisomes using these time constants was calculated to be 94%.

**Fig. 2:**
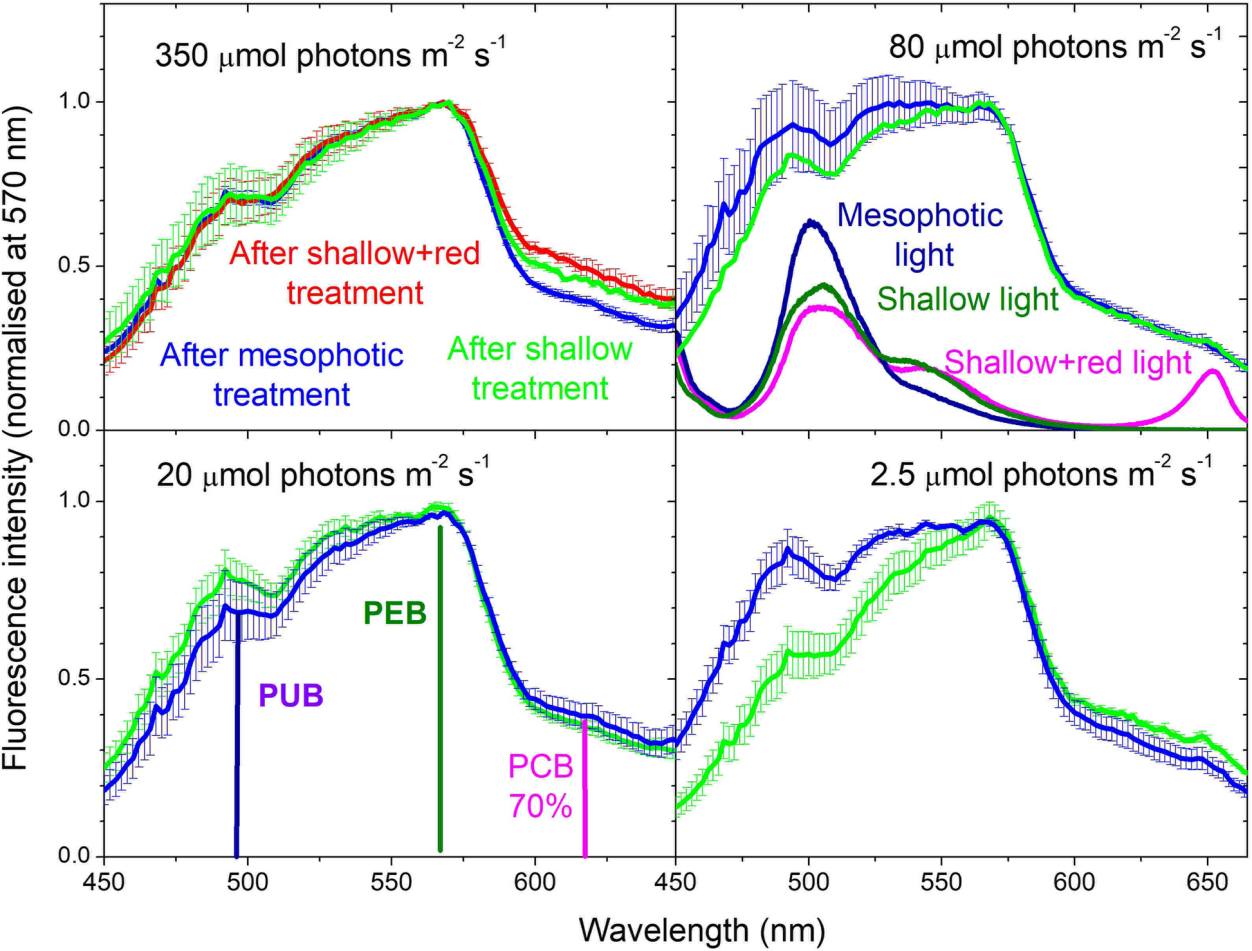
Excitation spectra of PSI fluorescence of *L. glaciale*. Detected at 720 nm (normalised at 570 nm) with a 15-nm bandwidth (full-width half maximum) after 9 months experimental exposure to different light intensities (350, 80, 20 and 2.5 µmol photons m^−2^ s^−1^) and spectra (shown for reference in the top right panel). Vertical lines indicate the wavelengths used to estimate the light harvesting contributions of phycourobilin (PUB), phycoerythrobilin (PEB) and phycocyanobilin (PCB). PCB accounts for only 70% of the indicated chlorophyll-*a* fluorescence intensity recorded after excitation at 620 nm, with the remaining 30% coming from the direct excitation of chlorophyll-*a* (see Additional File 1: Figure S1). Data presented as mean ± SE, *n* = 3–4 individuals (from different aquaria) per treatment

**Fig. 3:**
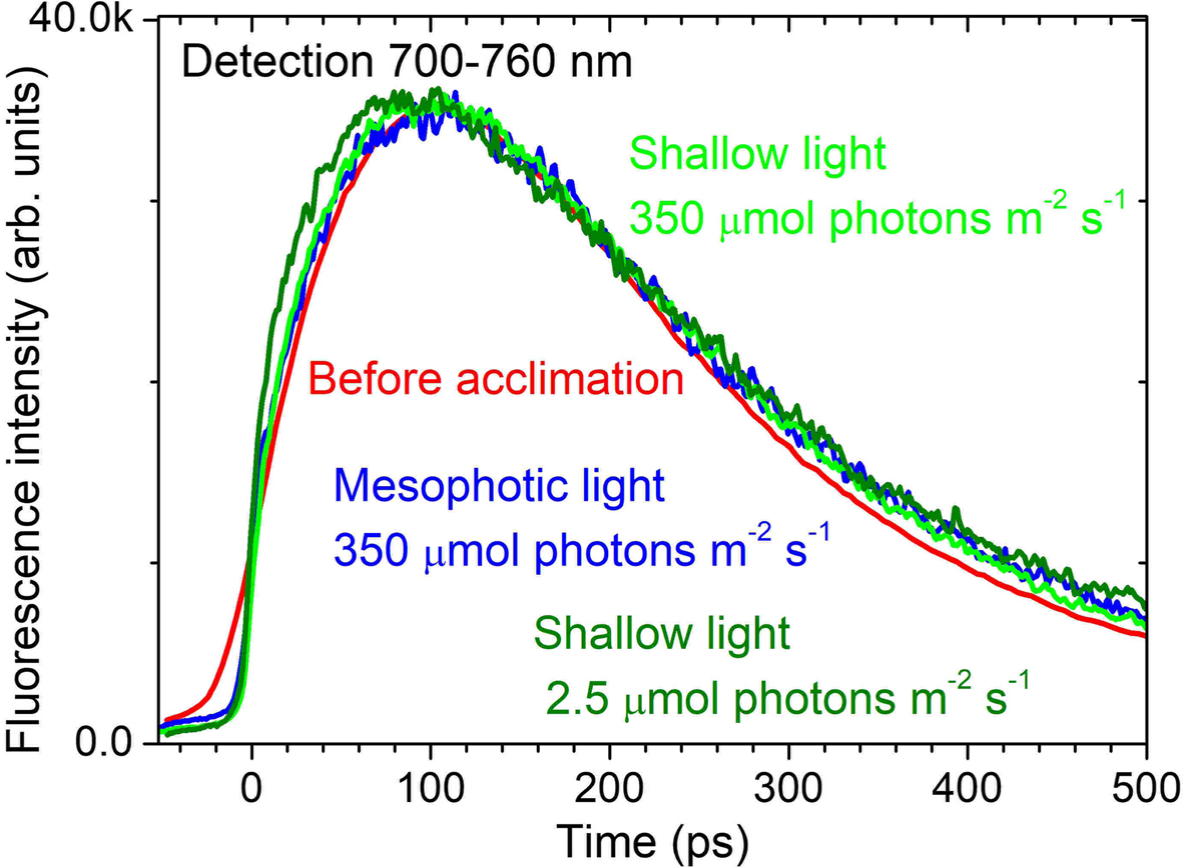
Representative fluorescence kinetics at 700–760 nm showing energy transfer to PSI after different light treatments

Having established the energy transfer baseline, we then used laboratory experimentation to identify the photo- and chromo-acclimation capacity of the *L. glaciale*. We maintained algal specimens under one of three different spectra, and a range of light intensities for 9 months. The three spectra were representative of one mesophotic and two shallow-water scenarios to compare the effects of a narrow vs broad light spectrum respectively (Additional File 1: Figure S3); light intensity levels were: 350, 80, 20 and 2.5 µmol photons m^−2^ s^−1^ PAR (see the ‘Methods’ section for full experimental details). Excitation spectra of PSI fluorescence indicate that excitation responses were different between spectral treatments at the lower three light intensities, but not at the highest light intensity (Fig. 2). Despite this apparent light harvesting interaction between light intensity and spectral treatment, no detectable change in energy transfer rate to PSI was observed, as revealed through the fast rise of chlorophyll-*a* fluorescence within ~ 100 ps, recorded at 700–760 nm (Fig. 3, Additional File 1: Figure S4). Energy transfer from peripheral rods of phycobilisomes to photosystems remained more than 90% efficient when acclimated to low light.

### Acclimation of *L. glaciale* phycobilisome composition to mesophotic light

**Fig. 4:**
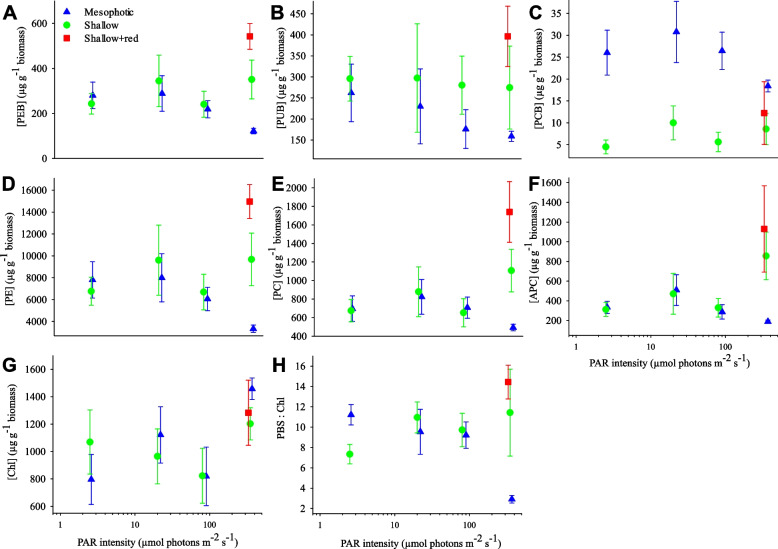
Bulk chromophore/phycobilin concentrations in *L. glaciale* under different light spectral compositions and PAR intensities after 9 months of laboratory incubation. **A** phycoerythrobilin (PEB), **B** phycourobilin (PUB), **C** phycocyanobilin (PCB), **D** phycoerythrin (PE), **E** phycocyanin (PC), **F** allophycocyanin (APC), **G** chlorophyll-a (Chl), and **H** ratio between total phycobilisome and chlorophyll-*a* concentrations (PBS:Chl). Data presented as mean ± SE, *N* = 4 individual algae per treatment. Data points are jittered horizontally around each experimental light intensity for visual clarity. Shallow + red spectrum tested at 350 µmol photons m^−2^ s.^−1^ only. Statistical model test results of relationship between each measured parameter and light intensity/spectral treatments are provided in Additional File 1: Table S1

Despite no apparent change in energy transfer rate to PSI, significant differences in phycobilisome composition were observed. A significant spectrum effect was observed for PEB, PUB, PCB, PE and PC concentrations (*p* < 0.05, Fig. 4A–E, Additional File 1: Table S1). When averaged over light intensity, the shallow and mesophotic spectral treatments were not significantly different (Additional File 1: Table S1). However, when explored at the highest light intensity only (enabling investigation of the role of + red light exposure), the mesophotic spectrum was significantly different from shallow and shallow + red spectral treatments. No significant difference between the shallow and shallow + red treatments was observed for PEB, PUB, PCB, PE nor PC (Additional File 1: Table S1). A similar pattern was observed for APC concentrations (Fig. 4F), but no significant spectrum effect was statistically determined (*p* = 0.4; Additional File 1: Table S1). In contrast, for chlorophyll-*a* (which had a concentration ~ 10 × lower than PE), an intensity rather than spectrum effect was observed (Fig. 4G; Additional File 1: Table S1). Post hoc test results indicated that the highest light intensity was associated with a higher chlorophyll-*a* concentration compared to both the 80 and 2.5 µmol phonos m^−2^ s^−1^ treatments (Additional File 1: Table S1). For the phycobilisome:chlorophyll-*a* concentration ratio (PBS:Chl), a significant spectrum × intensity interaction was observed (Fig. 4H; Additional File 1: Table S1). At the highest light intensity, PBS:Chl under a mesophotic spectrum was significantly different to both the shallow and shallow + red spectra (*p* < 0.05, Additional File 1: Table S1). No difference between the shallow and shallow + red spectra was observed (*p* = 0.3, Additional File 1: Table S1). A spectrum effect was not observed at the lower light intensities (Additional File 1: Table S1). Across levels of intensity and within levels of spectrum, the highest light intensity treatment was associated with lower PBS:Chl under a mesophotic spectrum (Additional File 1: Table S1).

**Fig. 5:**
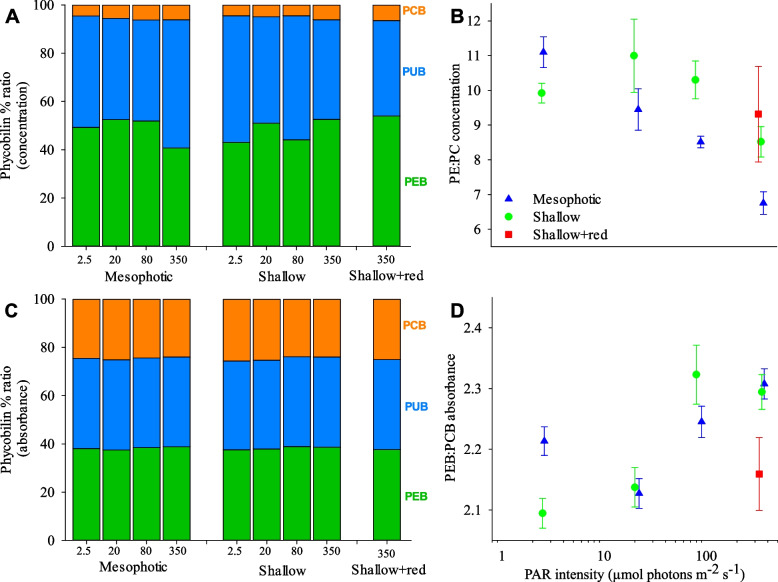
Chromophore and phycobilin composition and function in *L. glaciale* under different light spectral compositions and PAR intensities after 9 months of experimentation. **A** Proportional ratio between PEB, PUB and PCB concentrations (as % of their total concentration). **B** Ratio between phycoerythrin and phycocyanin bulk concentrations (PE:PC concentration). **C** Proportional ratio between PEB, PUB and PCB absorbances (as % of their total absorbance). **D** Ratio between PEB and PCB absorbance (PEB:PCB absorbance). Data in A/C presented as mean; B/D presented as mean ± SE. *N* = 4 individual aquaria per treatment. Data points in B&D are jittered horizontally around the experimental light intensity for visual clarity. Shallow + red spectrum at 350 µmol photons m^−2^ s.^−1^ only. Statistical model test results of relationship between each measured parameter and light intensity/spectral treatments are provided in Additional File 1: Tables S2 and S3

To further interrogate changes in the phycobilisome composition, we determined the ratio between PEB/PUB/PCB chromophore concentrations, between PE and PC bulk concentrations (PE:PC concentration), and between PEB and PCB spectral absorbance (PEB:PCB absorbance). PEB accounted for 41–54% of the total chromophore concentration, PUB 40–53% and PCB < 6.5% under all light intensities and spectra (Fig. 5A). A significant effect of spectrum was observed for PE:PC concentration (Fig. 5B), with post hoc test results indicating that PE:PC concentration was significantly lower in the mesophotic treatment compared to the shallow + red spectra (Additional File 1: Table S2). Although PUB and PEB concentrations varied with spectral treatment (Additional File 1: Table S1), their ratio was always close to 1:1 (Fig. 4A, B). Given a ratio of PUB absorbance at 495 nm to PEB absorbance at 560 nm of ~ 0.77 and its similarity to the ratio of chlorophyll-*a* fluorescence intensity observed under 495 nm excitation to that under 560 nm excitation (Fig. 2), this suggests negligible PEB contribution to light harvesting at 495 nm. This is consistent with the PEB extinction coefficient at 495 nm being much smaller compared to its peak [30]. PEB and PUB absorbances were ~ 12% higher than PCB (Fig. 5C). A significant interactive effect between light intensity and spectral composition on PEB:PCB absorbance and chromophore absorbance ratios was observed (Fig. 5D, Additional File 1: Figure S4, Table S3): At the highest light intensity, a divergence between spectral treatments was observed, characterised by higher PEB:PCB absorbance under a mesophotic spectrum, attributed to a reduced contribution by PC (Additional File 1: Figure S5; Table S3). No significant effect of the addition of red light into the shallow spectrum was observed (Fig. 5D, Additional File 1: Table S3).

### Acclimation of the *L. glaciale* light harvesting energy transfer to mesophotic light

**Fig. 6:**
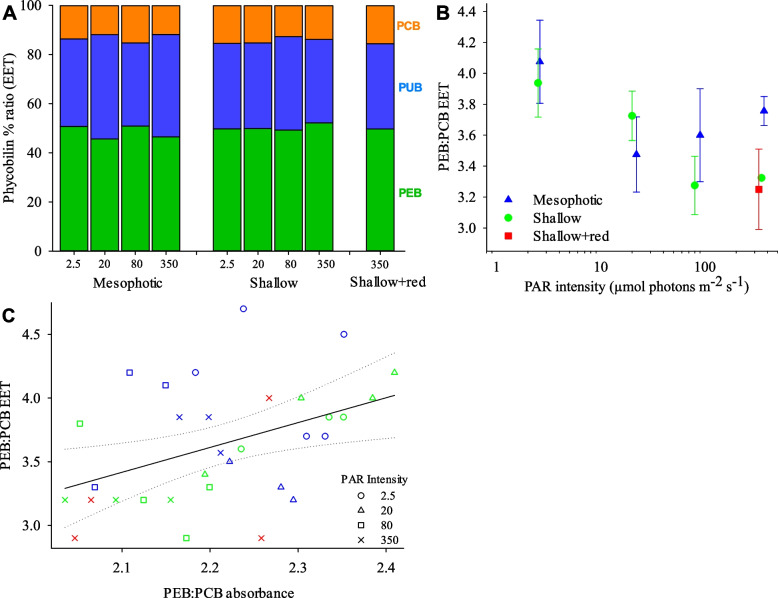
Phycobilisome excitation energy transfer in *L. glaciale* under different light spectral compositions and PAR intensities after 9 months of experimentation. **A** Proportional ratio between PEB, PUB and PCB EET (as % of their total EET). **B** PEB:PCB contributions to PSI fluorescence based on fluorescence excitation spectra detected at 720 nm (PEB:PCB EET). **C** Relationship between PEB:PCB absorbance and PEB:PCB EET; modelled linear regression (black solid line) with 95% confidence intervals (dotted lines) is shown: *y* =  − 0.679 + 1.95 × , *r*^2^ = 0.191, *p* = 0.012. Data in **A** presented as mean, and in **B** as mean ± SE where *N* = 4 individual algae (from different aquaria) per treatment (except mesophotic spectrum at 350 and 80 µmol photons m^−2^ s^−1^ where *N* = 3). Data points in **B** are jittered horizontally around the experimental light intensity for visual clarity. Shallow + red spectrum at 350 µmol photons m^−2^ s.^−1^ only. Statistical model test results of relationship between each measured parameter and light intensity / spectral treatments are provided in Additional File 1: Table S2

Chromophore-specific EET responses exhibited a significant interactive effect of light intensity and spectrum, characterised by a proportional increase in PUB compared to PEB and PCB under the mesophotic spectrum, particularly at 2.5 and 80 µmol photons m^−2^ s^−1^ light intensities (Fig. 6A, Additional File 1: Table S2). No significant difference in PEB:PCB EET was observed with light intensity nor spectral treatment (Fig. 6B, Additional File 1: Figure S5, Table S2). A significant positive linear relationship between PEB:PCB absorbance and PEB:PCB EET was observed (Fig. 6C).

### Acclimation of photosystems to mesophotic light

**Fig. 7:**
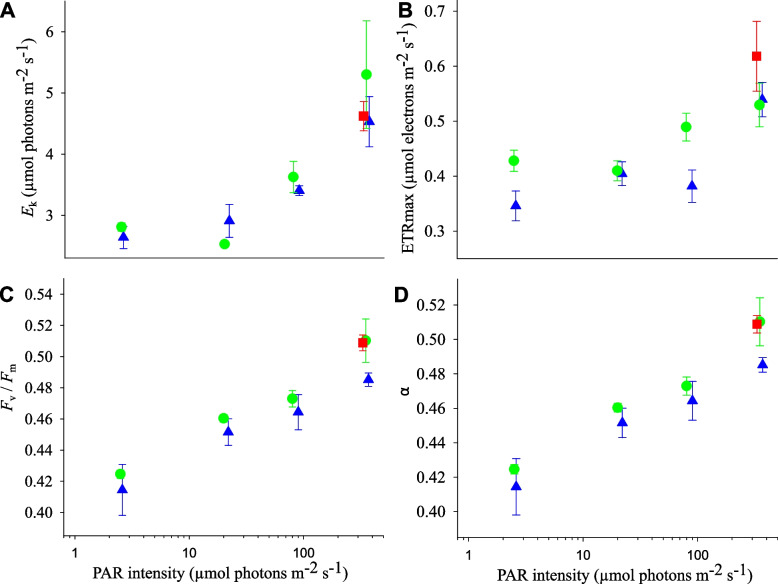
Photosynthetic characteristics of *L. glaciale* under different light spectral compositions and PAR intensities after 9 months of experimentation. **A** Minimum saturating irradiance (*E*_*k*_); **B** maximum obtained electron transport (ETR_max_); **C** Maximum quantum yield (*F*_*v*_/*F*_*m*_; no units) and **D** photosynthetic efficiency in light-limiting conditions (*α*; no units). Data presented as mean ± SE, *N* = 4 individual aquaria per treatment. Data points are jittered horizontally around the experimental light intensity for visual clarity. Shallow + red spectrum at 350 µmol photons m^−2^ s.^−1^ only. Statistical model test results of relationship between each measured parameter and light intensity/spectral treatments are provided in Additional File 1: Table S4

Pulse-amplitude modulated (PAM) fluorometry was used to test the effect of light spectrum and intensity on the functioning of PSII. A significant decrease in minimum saturating intensity (*E*_k_), and photosynthetic efficiency (as *F*_v_/*F*_m_ and α) was observed as light intensity decreased (Fig. 7, Additional File 1: Figure S6, Table S4). Similarly, maximum obtained electron transport rate (ETR_max_) showed a decreasing trend with increased light intensity (Fig. 7B), but this was only significantly different for the mesophotic spectrum (Additional File 1: Table S4). No significant difference between spectral treatments was observed for *E*_k_, *F*_v_/*F*_m_ nor α (Fig. 7; Additional File 1: Table S4). Posthoc test results indicated differences in ETR_max_ between the shallow and mesophotic spectra at the two highest light intensities (Fig. 7B, Additional File 1: Table S4). Temporal photoacclimation was observed over the course of the 9-month experiment, characterised by a significant reduction in *E*_k_ and ETR_max_ and an increase in *F*_v_/*F*_m_ and α over time (Additional File 1: Figure S6, Table S4).

## Discussion

Photo-acclimation to light intensity and chromo-acclimation to monochromatic light is known to occur across photosynthetic taxa. However, questions remain over the mechanisms behind these acclimatory responses, and mono-chromatic experimental investigations have limited ecological relevance. Here, we present the first, to our knowledge, energy transfer model of red coralline algae, and provide experimental evidence to causatively demonstrate how red algal light harvesting energy cascades initiate photo and chromo-acclimation in response to a mesophotic light intensity and spectrum. This detailed insight into photosynthesis under extreme low-light environments provides a photo-physiological pathway for enabling the success of red coralline algae in the mesophotic zone.

### Light harvesting in *L. glaciale*

The absorbance spectra indicate the presence of typical pigments found in Rhodophyta, supporting previous studies investigating red coralline algal pigments [e.g. 15, 31]. Phycobilisome pigments resulted in an especially strong light absorption in the mid-range of the visible light spectrum – an area where chlorophyll-*a* has extremely limited absorption. At a chromophore level, PEB, PUB and PCB were all responsive to changing light conditions. Energy transfer through the phycobilisome was rapid (~ 120 ps) – an order of magnitude faster than the natural decay time of PE in water (~ 1.5 ns, Additional File 1: Figure S2). Other cyanobacterial systems have reported comparable energy transfer times [29], whilst some are slower [32, 33] and others faster [34].

This rapid energy transfer is perhaps achieved because of plasticity in the energy transfer pathway that enables direct transfer from PE to PSI/PSII, effectively bypassing the other phycobilisome constituents (PC and APC) if necessary. This suggests that phycobilisomes of *L. glaciale* might contain rather short rods of PE and PC hexamers (Fig. 1), which may allow for improved flexibility in the phycobilisome spatial arrangement along the thylakoid membrane. This acclimatory poise appears to be driven primarily by the chromophores PEB and PCB; PUB-guided energy transfer did not respond to light intensity nor spectral treatments. Previous reports on red algal phycobilisome structure have found that phycobilisome rods can vary between PE / PC composites and solely PE (plus linker proteins) [21, 27]. High-resolution phycobilisome structures in the red algae *Griffithsia pacifica* and *Porphyridium purpureum* [21, 27] indicate that PE rods can be found directly next to the phycobilisome core. Our results indicate that this enables direct energy transfer from PE to APC, establishing a light harvesting pathway that would be a particularly efficient strategy in extreme low light (e.g. in the mesophotic). Since bulk chlorophyll-*a* concentration was about ten times lower than the PE, energy transfer to both photosystems is required for equal excitation and is essential for maintaining a balanced linear flow of electrons. Here, we show that this flexibility in both rod design and in parallel transfer to both PSI and PSII enables near-unit efficiencies—comparable to that observed in purple bacteria [35]. Importantly, our controlled experimental approach causatively shows that this can be maintained under varying light spectra. The large distribution of pigment energies and scattering of emitted light by the complex skeleton of the living red coralline algae prevented us from distinguishing between PSI and PSII energy transfers. Here, we consider the effects of long-term change (months), but pathways may be different at shorter time scales. The dark–light transition, for example, is managed via reversible phycobilisome decoupling from PSI without subsequent attachment to PSII [34]. Thus, in order to gain a complete picture of acclimatory capacity, it must be considered across the full range of ecologically-relevant temporal scales. For red coralline algae, this might be from a diel to centennial timeframe, to reflect natural daily-seasonal transitions and longer-term climatic transitions experienced during their decadal-centennial lifetime.

### Acclimatory mechanisms to low light intensity

All specimens remained alive at the end of the experiment, demonstrating that, although at the threshold for photophysiological limitation, 2.5 µmol photons m^−2^ s^−1^ was not limiting for the survival of *L. glaciale*. Results from this study demonstrate that two key strategies are adopted to maintain photosynthesis under low light intensities.

Firstly, modification of the phycobilisome composition and function enabled energy transfer rates to be maintained regardless of light intensity. Despite the observed increase in PE:PC contribution to light harvesting in response to low light intensity — attributed to a proportional increase in PE content at the expense of PC — the rate of EET was not affected. This, together with the overall EET rates observed, indicates that excitation energy is quickly and efficiently transferred to the photosystems via a stable phycobilisome core and does not represent a bottleneck in energy supply, even under low light intensities. Compositional change in the phycobilisome rods (i.e. PE:PC ratio) under low light increases absorption of the available blue and green light and hence the light harvesting capacity of phycobilisomes. This can be further modulated within the phycobilisome core, where linker proteins can fine-tune the red-shifted absorbance of the bound PCB [21]. The phycobilisome structure therefore provides a complex light harvesting system that is highly responsive to incoming light conditions. We do not expect this to negatively impact the ‘packaging effect’ (i.e. intracellular pigment shading) because of compensatory mechanisms (e.g. [36]) and the thin photosynthetically active layer of red coralline algae relative to their thallus size. Alterations in chromophore/phycobilin composition may also support other functions such as photoprotection or nutrient storage [37]. However, the positive relationship between PE:PC absorbance and EET found in this study suggests that phycobilin modification is utilised here to optimise energy supply to the photosystems. This suggests the observed PE upregulation is associated with phycobilisome modification, rather than occurring as ‘free’ biliproteins within the stroma [37]. Of course, to enable an increase in PE concentrations, there must be a transcriptional upregulation of the genes associated with PE biosynthesis, including *peA* and *peB* (for the synthesis of the PE α and β protein subunits [38]), and *pebA* and *pebB* to catalyse the conversion of biliverdin IX α to PEB via two two-electron reductions [39]. Characterisation of their expression patterns under each experimental treatment would have further resolved the biochemical acclimation pathway and provided additional insight into the physiological balances required to maintain energy transfer.

Secondly, acclimation of PSII in response to light intensity was also observed, including a reduction in the optimum light requirements (*E*_k_) and electron transport rates (ETR_max_). This allows for more efficient photosynthesis, supporting previous work investigating experimental [14, 16, 40, 41] or natural [17, 42–45] reductions in irradiance. However, in contrast to these previous studies, we did not observe a concomitant increase in photosynthetic efficiency at low light intensities. When environmental conditions become challenging, a plant’s ability to maintain α is inhibited [46, 47]. Under this study’s light intensities, light levels were generally at or above *E*_k_, indicating a perpetual state of light saturation during daytime hours and therefore a need for prolonged photoprotection measures. Under these conditions, red coralline algae are known to exhibit reduced photosynthetic efficiency as a photoprotective strategy [15].

### Acclimatory mechanisms to mesophotic light spectra

At the lowest light levels, acclimatory responses were dominated by available light intensity. However, spectral effects were also observed, indicating the capacity for chromo-acclimation. Although not directly measured here, photoreception in *L. glaciale* is likely mediated by blue-light-induced conformational changes to the C-terminus of cryptochrome receptors derived from DNA photolyases [48]. Other photoreceptor families such as phototropins and phytochromes do not appear to be present in red algae [49, 50]. In the high-intensity mesophotic treatment, there was a higher availability of high-energy blue-green light. PEB:PCB EET was consistently high at all light intensities under the mesophotic spectrum, reflecting an increase in the energy of the supplied photons. Interestingly, this did not appear to be due to an increase in the PUB:PEB ratio (i.e. type IV chromatic acclimation), which would require an upregulation of bilin lyase isomerases (e.g. MpeV [51]) for PEB to PUB conversion. Instead, *L. glaciale* appears to prioritise type II and III chromatic acclimation to induce PE/PEB production over PC/PCB (which were an order of magnitude lower in concentration) [52]. However, the consistency of energy transfer rates to PSI/PSII across treatments suggests that this was tempered by photophysiological acclimation. The high-power mesophotic spectra pose an increased risk of photo-damage compared to the broadband spectral availability in ‘shallow’ spectra, necessitating an increase in photoprotective strategies – evidenced here as reduced light harvesting capacity via reduced phycobilisome pigmentation. Under the highest intensity mesophotic treatment, PEB and PUB concentrations were significantly lower, perhaps indicative of an active photoinhibition strategy to limit energy absorption and minimise photodamage. Phycoerythrin may have also been decoupled from the phycobilisome for photoprotection [53, 54], effectively reducing light harvesting-active PE despite an apparent increase in PEB:PCB absorbance. At lower light intensities under the mesophotic spectrum, an increase in PE would be preferable to optimise harvesting of the available photon wavelengths. Phycobilisome acclimation to a mesophotic light regime therefore reflects a balance between photon availability and energetic cost. PSII acclimation to the mesophotic spectral regime was also observed over time, with features that typify low-light optimisation including a reduction in optimal light requirements and electron transport rates and an increase in photosynthetic efficiency. This effect was accentuated under higher light intensities.

Although chlorophyll-*a* can directly absorb red light (the Qy band; [55]), no discernible acclimatory response was observed when red wavelengths were available. Due to rapid attenuation of red light in seawater (Kirk, 1984), sub-tidal red coralline algae are unlikely to be exposed to appreciable levels of red wavelengths in the natural environment. Evolutionary adaptation has resulted in the use of spectral photosynthesis by red algae [56]. In support of this, we observed no difference in photosynthetic pigmentation when red light wavelengths were available (although this spectral treatment was characterised by a generally higher variability), and that the blue- and green-light-absorbing PUB and PEB contributed more than 80% to the chlorophyll-*a* fluorescence intensity, indicating that red light plays a limited role in the photosynthetic functioning of *L. glaciale.*

## Conclusions

Despite the widespread occurrence of photosynthetic organisms throughout the oceanic mesophotic zone, how photosynthesis can proceed in such a low light environment remains unclear. Here, we provide mechanistic insight into the photo- and chromo-acclimatory capacity of the red coralline alga *L. glaciale* to ecologically relevant limitation in both light intensity and spectral availability. Our results demonstrate that, following photon absorption, *L. glaciale* can actively progress photon energy transfer an order of magnitude faster than the natural rate of energy decay in PE. Energy transfer rates are optimised through modification of the phycobilisome construction, utilising both a two-stage and three-stage energy cascade and ensuring delivery of energy to both photosystems to maximise photosynthetic turnover. Light intensity, rather than spectral composition, is the dominant factor in driving acclimatory responses, supporting coralline algal presence in shallow-water low light environments such as caves, at high latitudes and under macrophytic canopies. This indicates that efficient photon harvesting, rather than spectral optimisation, may be key for red coralline algal mesophotic photosynthesis. By increasing the breadth of light harvesting across the visible light spectrum beyond that of chlorophyll-*a* absorption, we show the importance of phycobilisomes in enhancing light harvesting capacity to ensure photosystem energy supply is maintained. Flexibility in light harvesting strategies is therefore key to explaining red algal and cyanobacterial photosynthesis at the lower limit of the oceanic mesophotic zone.

## Methods

### Experimental specimens

Free-living red coralline algae (*Lithothamnion glaciale*) were hand collected using SCUBA from 6 m depth at Loch Sween, west coast of Scotland (56°01.99′N, 05°36.13′W) (total *n* = 360). Thalli were stored in 100 L dark containers filled with local seawater and transported to the Wolfson Research Aquarium at the Lyell Centre, Edinburgh, within five hours of collection. Thalli were transferred to the laboratory aquariums (*n* = 10 per aquarium; 28 L water capacity) and maintained at ambient conditions for a two-week acclimatisation period. Following this, the light environment of each aquarium was randomly assigned an experimental treatment regime.

### Experimental treatments

Both light intensity and light spectrum were considered in a crossed design, enabling individual and interactive effects of environmentally-relevant conditions to be determined. The experiment was run for 9 months (June 2019–March 2020; COVID-19 lockdowns prevented the experiment from continuing). Measurements of algal light absorbance and PSII characteristics were taken at 3, 6 and 9 months. EET measurements were taken at 3 and 9 months. At the end of the experimental period, all algal samples remained alive with no visible signs of bleaching or necrosis.

For light intensity, there were four PAR treatment levels: 350, 80, 20 and 2.5 µmol photons m^−2^ s^−1^ photosynthetic photon flux density (PPFD), equivalent to 15, 4, 1 and 0.1% of a surface irradiance of 2000 µmol photons m^−2^ s^−1^ PPFD. Light intensities were achieved by layering neutral density (ND) filters (Rosco) above the aquaria and below the light source. The highest PAR treatment (350 µmol photons m^−2^ s^−1^ PPFD) used one 209 0.3ND (51% transmission) filter. Light intensity treatments 80–2.5 µmol photons m^−2^ s^−1^ PPFD had one, two and three layers respectively of the 211 0.9ND (13% transmission) filter.

For light spectra, there were three treatments, representative of the spectral composition of mesophotic and shallow euphotic environments (Additional File 1: Figure S3). The ‘mesophotic’ treatment had reduced wavelengths > 550 nm and an elevation in mid-range wavelengths (λ_max_ 495 nm). The ‘shallow’ treatment consisted of a broadly uniform distribution of wavelengths from 400 to 600 nm. The ‘shallow + red’ treatment was the same, with the addition of a long-wavelength red peak (λ_max_ 665 nm) to simulate the known presence of red light in the ocean’s surface waters. Shallow and mesophotic treatments were tested at all light intensities. To maintain the environmental relevance of the experimental treatments, the shallow + red spectrum was tested at 350 µmol photons m ^−2^ s^−1^ PPFD only since red light is quickly attenuated in the ocean. Algae exposed to red light would thus only experience this under high light intensity conditions. Light spectra were achieved through custom-designed aquarium lamps with four intensity-adjustable channels of six LEDs each (LEDZEAL). A 3-mm diffuser (CutMyPlastic) was positioned above the aquaria to ensure an even distribution of light intensity and spectrum throughout the aquaria.

Water changes were conducted weekly with 30 µm filtered seawater; salinity was monitored daily and maintained at 32–34 through freshwater dilution as required. Water temperature ([57] and unpublished data from Burdett) and day/night cycles (obtained from www.timeanddate.com) were maintained at ambient in situ levels throughout the experiment.

### Algal chromophore/phycobilin concentration

At the end of the experiment, one sample (~ 0.2 g) per aquarium was quantified for chlorophyll-*a*, PE, PC, APC, PUB, PEB and PCB concentrations. Samples were rinsed in Milli-Q water and immediately stored at -80 °C until analysis. Pigments were extracted in 90% acetone (chlorophyll-*a*) or 0.1 M phosphate buffer at pH 6.8 (phycobilisome pigments) via ice-cold ultrasonication in the dark. Pigment concentrations were determined spectrophotometrically (Biowave3, BioChrom) using the protocol and modified equations of Connan [58]. Native extinction coefficients (ε) for phycobilin concentration calculations were: 105,000 M^−1^ cm^−1^ for PUB, 136,000 M^−1^ cm^−1^ for PEB [30] and 102,000 M^−1^ cm^−1^ for PCB [59] (*M*_*r*_ = 587 g mol^−1^ for all).

### Algal light absorption

Optical reflectance of the surface of each individual was measured as tissue chromophore/phycobilin composition following an adapted method from Burdett et al. [15]. A Flame-S-VIS–NIR Miniature Spectrometer (Ocean Optics) was used, equipped with a high-power tungsten halogen light source (HL-2000-HP-FHSA; 360–2400 nm) and 400 mm lab-grade reflection probe (Ocean Optics). An integration time of 2500 ms was used with a boxcar width of one. The spectrometer probe was fixed at a 45° angle for all measurements to ensure the measurement of diffuse reflectance and avoid specular light effects that can occur when the probe is perpendicular to a sample. Following the manufacturer’s recommendation, the probe was held 4 mm from the algae to maximise the efficiency of the overlapping illuminating fibres. The spectrophotometer was calibrated using a white reflectance standard (Ocean Optics) that represents 100% reflectance and a ‘dark’ calibration to account for any ambient light before each set of measurements (measurements were conducted in the dark to minimise external light interference). Absorbance was calculated using the formula [60, 61]:

$$D\left(\lambda \right)=\mathrm{log}\left[1/R\left(\lambda \right)\right]$$

where *D* = absorbance, *λ* = wavelength and *R* = reflectance. Absorbance was corrected for non-pigment absorption by subtracting the average absorbance between 750 and 800 nm. Chromophore-specific maximum absorbance peaks were used as a proxy for relative chromophore abundance: Chlorophyll-*a* (680 nm), PC (630 nm) and PE (565 nm). At 590–640 nm, there is an overlap between chlorophyll-*a* (30% contribution) and PC (70% contribution) (Additional File 1: Figure S1). Thus, a 0.7 correction factor was applied to the PC absorbance. Ratio between PEB and PCB absorbance (PEB:PCB absorbance) is presented.

### Photosystem characteristics

To investigate the characteristics of photosystem function, chlorophyll-*a* fluorescence measurements (> 640 nm detection) were conducted using PAM fluorometry with a blue-light Junior PAM (Walz GmbH). Samples were dark adapted for 30 min prior to analysis. Measurements were taken following previous methodologies and notations [15, 62]. A 2-mm-diameter fibre optic probe was used for all measurements, positioned on the algal tissue. PAM settings were: measuring light intensity = 8, gain = 1, saturation pulse intensity = 12, saturation pulse width = 0.6, actinic light intensity = 5. The excitation light of the blue-light Junior PAM has a maximum of around 445 nm with a full-width half maximum of 23 nm. Fluorescence is detected at > 640 nm. Although this excitation light excites phycobilisomes as well as chlorophyll-*a*, the observed changes in pigment ratios could cause only < 5% change in photosynthetic efficiency *F*_v_/*F*_m_: the excitation light of the blue-light Junior PAM has a maximum of around 445 nm with a FWHM of 23 nm. Fluorescence is detected at > 640 nm and includes phycobilisome fluorescence at 630–670 nm and chlorophyll-*a* fluorescence at > 670 nm. Where phycobilisomes absorb fraction *b* of the excitation light, phycobilisome fluorescence *C* does not change with the saturating flash, whilst chlorophyll-*a* fluorescence changes from *F*_0_ to *F*_m_. PAM measured signals are therefore:

$${f}_{0}=b({F}_{0}+C)+(1-b){F}_{0}={F}_{0}+bC$$

And

$${f}_{m}=b({F}_{m}+C)+(1-b){F}_{m}={F}_{m}+bC$$

And apparent ‘dark efficiency’ is therefore:

$$\frac{{f}_{v}}{{f}_{m}}=\frac{{f}_{m}-{f}_{0}}{{f}_{m}}=\frac{{F}_{m}-{F}_{0}}{{F}_{m}+bC}$$

Fluorescence spectra in Additional File 1: Figure S1A suggest that *bC ≈ F*_*m*_. The fraction *b* is proportional to the PE:Chl concentration ratio, which changes < 10% with different treatments (Fig. 2A and B). This causes < 5% change in the apparent ${f}_{v}\left/{f}_{m}\right.$.

Rapid Light Curves (RLCs) were used to provide information on energy dissipation from light-limiting through to light-saturating conditions, representing actual rather than optimal photosynthetic state [63]. RLCs used eight irradiance steps each of 10-s duration ranging from 0 to 420 µmol photons m^−2^ s^−1^. The minimum saturating intensity (*E*_k_ — the irradiance level at which light shifts from being photosynthetically limiting to photosynthetically saturating; units = µmol photons m^−2^ s^−1^) and initial photosynthetic rate (alpha [α]; no units) were calculated by fitting RLC data to the irradiance-normalised nonlinear least squares regression model of Jassby and Platt [64] in the R package Phytotools [65]. All model fits were statistically significant (model *p*-value < 0.0001 for all). Maximum obtained ETR (ETR_max_) values from the RLCs are based on electron transport rate (ETR, µmol electrons m^−2^ s^−1^) calculated from *F*_q_′/*F*_m_′ measurements at each actinic light intensity (E) of the RLC:

$$\mathrm{ETR}={F}_{q}^{^{\prime}}/{F}_{m}^{^{\prime}}\times \mathrm{PAR}\times 0.15\times A$$

where PAR = PAR levels of each RLC step, 0.15 is a multiplication factor to take into account that ~ 15% of chlorophyll-*a* in red algae is associated with PSII [62, 66–68] and *A* is the corrected total algal absorbance. The fraction of light absorbed by photosynthetic pigments (*A*) was calculated following Schubert et al. [69]:

$$A=1-{10}^{-D}.$$

where *D* is the average absorbance between 400 and 700 nm (= range of PAR), determined from the algal absorbance measurements (method described above).

### Excitation energy transfer (EET)

After 3 and 9 months of laboratory incubation, a branch from one randomly chosen individual per aquarium was collected, individually stored in plastic bags filled with seawater (250 ml) and transported in the dark at experimental temperature to the photonics laboratory at the University of St Andrews. Time-resolved fluorescence spectra were measured within 2 days of sampling using a Hamamatsu streak camera in synchroscan mode at room temperature. The samples were excited using 200 fs laser pulses at 515 nm and 80 MHz repetition rate with an average laser power density of about 50 mW cm^−2^. Fluorescence was detected at slightly less than 90° to excitation to minimise reabsorption and to maintain high time resolution. Time-resolved fluorescence spectra were globally analysed with a kinetic model of consecutive energy transfer between three compartments (A → B → C) using CarpetView software from Light Conversion. The spectral amplitudes of each compartments and time constants of transfer between them were adjusted to get randomly distributed residuals between the experimental and model spectra.

Fluorescence excitation spectra were measured within 12 h of sampling using a FLS980 spectrometer from Edinburgh Instruments. PEB:PCB EET ratio was determined from the spectral intensities at 565 nm (PEB) and 620 nm (PCB — with a 0.7 correction factor applied, as above). Light harvesting efficiency by phycobilisomes was estimated from the energy transfer time constants, as 120^−1^/(120^−1^ + 1500^−1^). Assuming PEB and PUB have similar absorption strength per mass, we estimate that the PUB contribution to light harvesting at 495 nm is ~ 50% (based on the PUB:PEB ratios for concentration [~ 0.4], absorbance and EET [both ~ 0.8]).

### Statistical analyses

All statistical analyses were performed in R version 3–5.1 [70] and presented in Additional File 1: Tables S1-S4). The effects of light intensity (four levels, considered a fixed factor) and spectral composition (three levels, considered a fixed factor) on PE:PC concentration and PEB:PCB EET and the relationship between PEB:PCB absorbance and PEB:PCB EET were analysed via a type-II Anova using the ‘car’ package and the ‘emmeans’ package for posthoc comparisons using Tukey adjustment [71, 72]. Model assumptions of normality and homogeneity of variances were tested using residual plots—data that did not meet these assumptions were transformed as required. To determine the effects of light intensity, spectral composition, and time (three levels, considered a fixed factor to account for the non-continuous variation in temperature and daylength over the sampling period) on PEB:PCB absorption and photosystem characteristics, linear mixed effects models were created using the ‘lme4’ package [73] and analysed with Anova type-III tests. To account for repeated measurements across time, ‘aquarium’ was added to the model as a random effect with uncorrelated random intercept and random slope. The effect of the random variable is not included in the test results output tables (see Additional File 1). *P*-values for post hoc comparisons use Kenward-Roger approximation for the degrees of freedom and with Tukey adjustment using the ‘emmeans’ package [72]. All analyses were based on aquarium averages (*n* = 4 aquaria, 10 individuals per aquarium), apart from the pigment concentration and EET analyses, where one randomly chosen sample per aquarium was used.

## Supplementary Information

**Additional** **file** **1.**

**Additional file 2.**

## Data Availability

The datasets supporting the conclusions of this article are included within the article and its additional files.
