# Supplementary material for: Red algae acclimate to low light by modifying phycobilisome composition to maintain efficient light harvesting
Source: BMC Biol. 2022 Dec 27;20:291. doi: 10.1186/s12915-022-01480-3 (PMC9794408; doi:10.1186/s12915-022-01480-3)
Supplement: Supplementary file 1 — Additional file 1. [file 12915_2022_1480_MOESM1_ESM.pdf]

Additional File 1

Red algae acclimate to low light by modifying phycobilisome composition to maintain efficient light harvesting

Sofie E. Voerman, Arvydas Ruseckas, Graham A. Turnbull*, Ifor D. W. Samuel*, Heidi L. Burdett*

*Co-corresponding authors: gat@st-andrews.ac.uk; idws@st-andrews.ac.uk; h.burdett@hw.ac.uk

Includes:

Figure S1-S5

Tables S1-S4

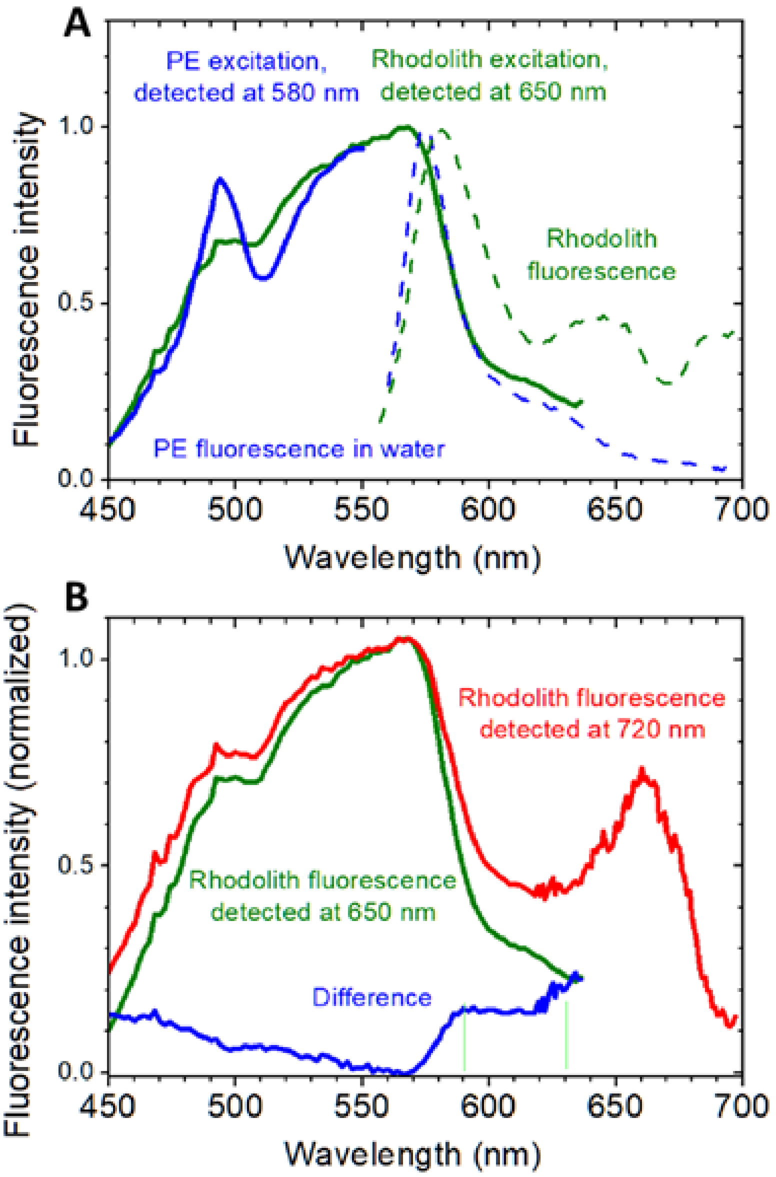

Figure S1. Fluorescence and fluorescence excitation spectra of *L. glaciale*. (***A***) Representative fluorescence (dashed lines) and fluorescence excitation spectra (solid lines) *L. glaciale* and of phycoerythrin (PE) in deionized water. (***B***) Excitation spectra of PSI fluorescence at 720 nm (red line) and phycocyanin fluorescence at 650 nm (green line) in *L. glaciale*, and the difference between them (blue line). The difference at 570-620 nm is attributed to absorption by chlorophyll *a* in PSI which shows the main peak at 662 nm and its higher vibronic band overlaps with phycocyanin absorption. The difference spectrum makes about 30% of the PSI excitation intensity at 590-620 nm; 70% can thus be attributed to phycocyanin.

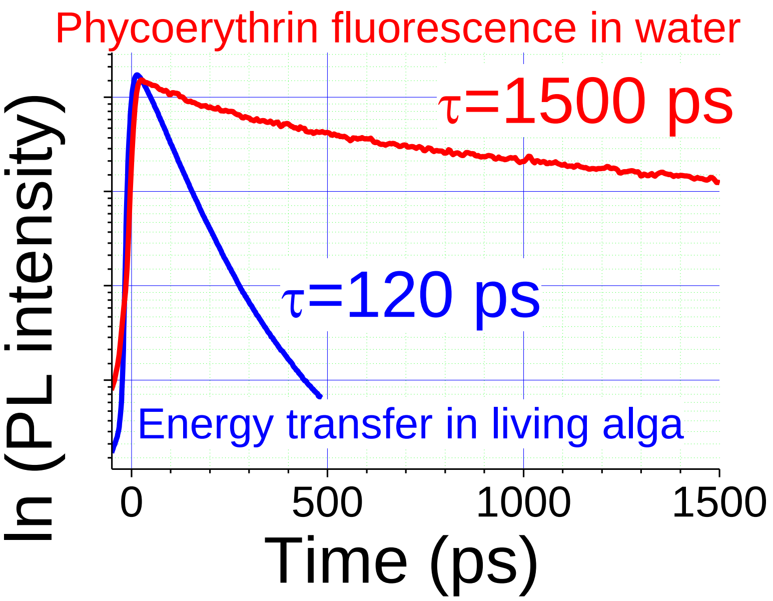

Figure S2. Phycoerythrin fluorescence decay in *L. glaciale* (with an energy transfer time of 120 ps) (blue line) and the natural decay in water (exciton lifetime of 1500 ps).

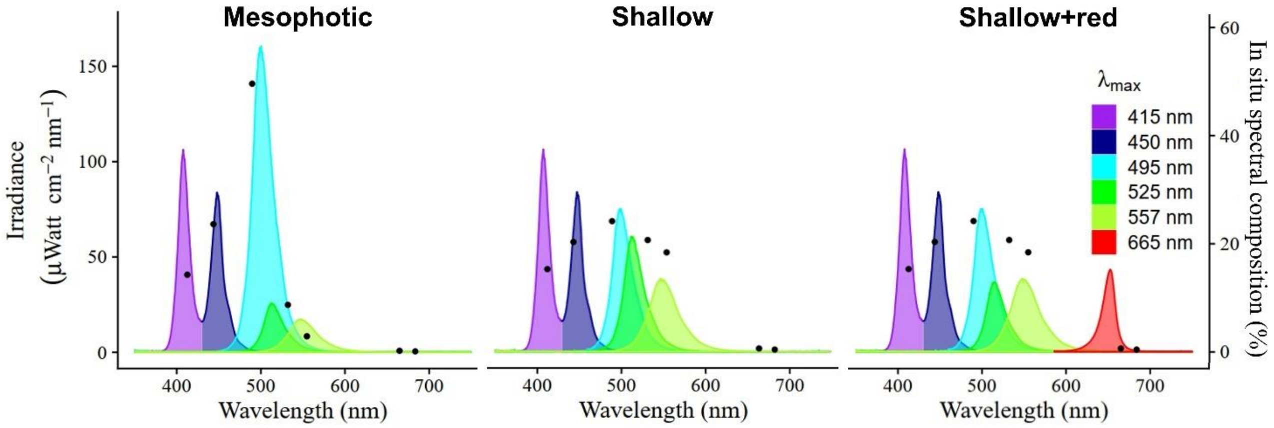

Figure S3. Spectral composition of the mesophotic, shallow and shallow+red spectral experimental treatments (coloured shading). Black dots indicate actual measured in situ spectral compositions by the authors from ocean archipelago mesophotic waters (86 m depth) and shallow coastal euphotic waters (13 m depth) (Voerman et al, in review).

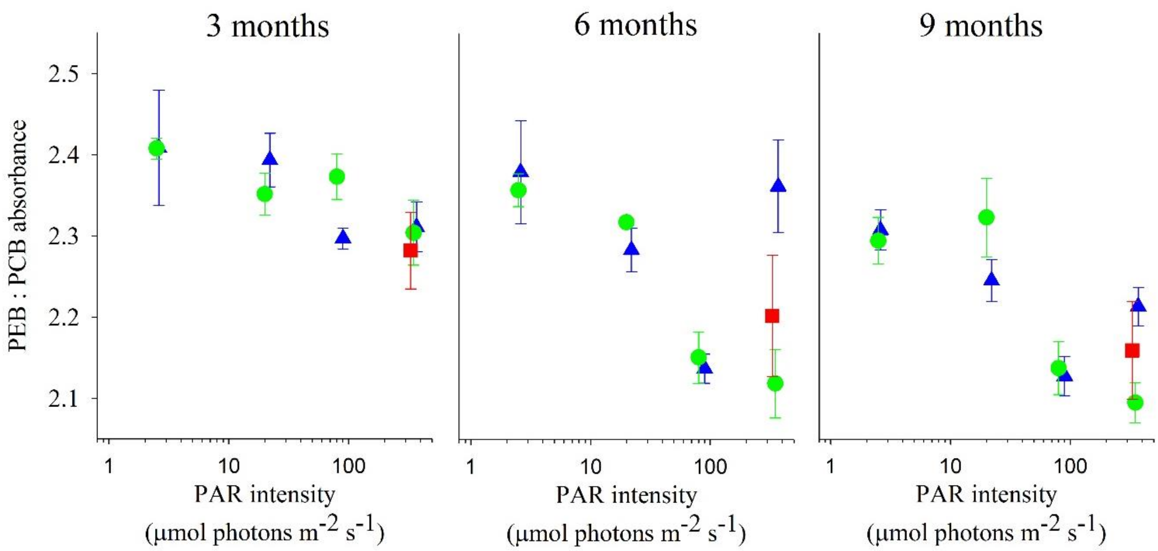

Figure S4. PEB:PCB absorbance of *L. glaciale* grown under different light spectral composition and intensities after three, six and nine months incubation. Data presented as mean±SE, N = 4 individual aquaria per treatment. PEB: phycoerythrobilin. PC: phycocyanobilin. Data points are jittered horizontally for visual clarity. Shallow+red spectrum at 350 μmol photons m^{-2} s^{-1} only.

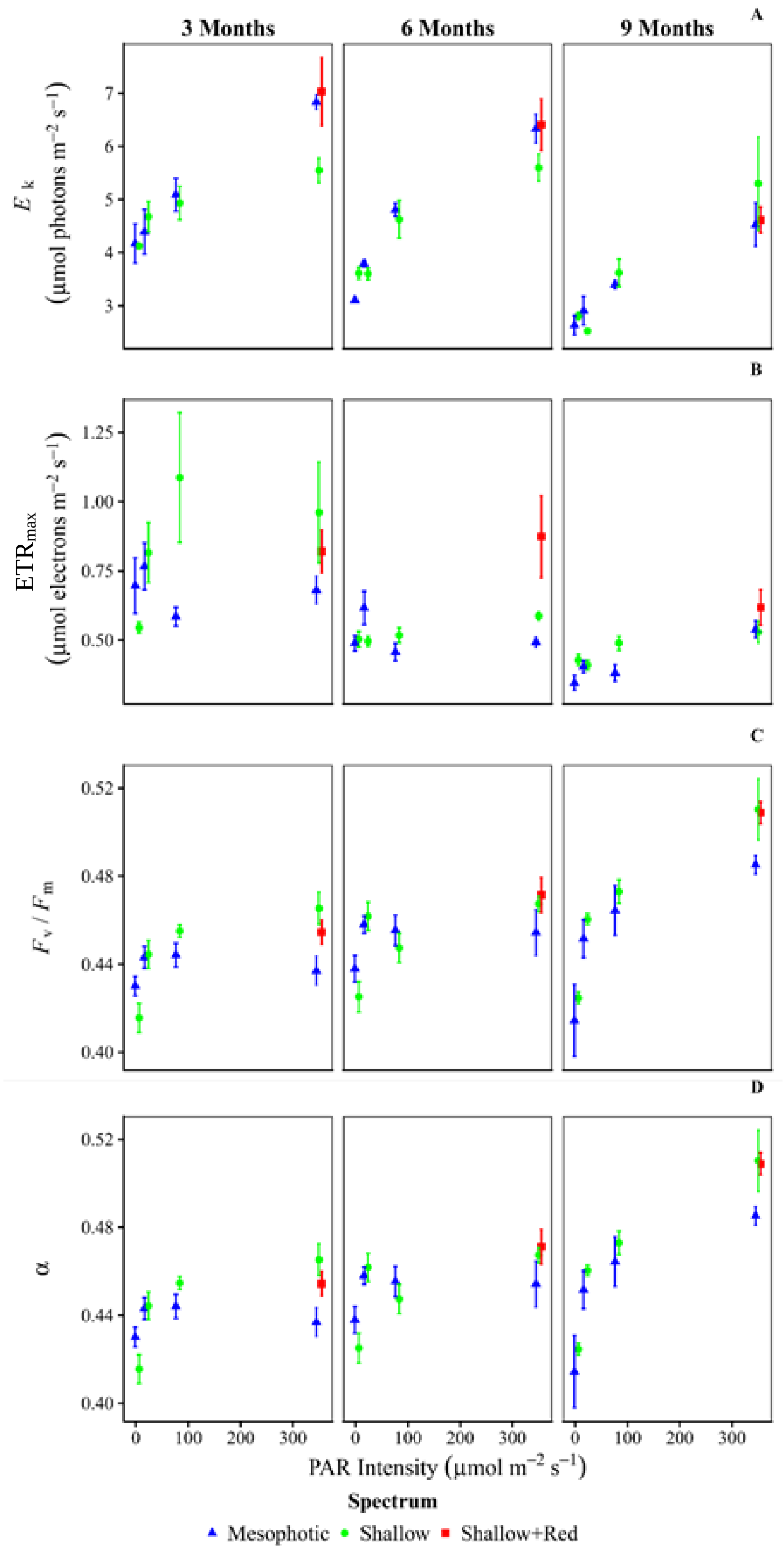

Figure S5. Photosynthetic characteristics of *L. glaciale* under different light spectral compositions and intensities after three (left), six (centre) and nine (right) months of experimentation. **A)** Minimum saturating irradiance (E_k); **B**) maximum obtained electron transport (ETR_{max}); **C**) Maximum quantum yield (F_v/F_m) and **D**) photosynthetic efficiency in light-limiting conditions (α). y-axes for C and D: arbitrary units. Data presented as mean±SE, N = 4 individual aquaria per treatment. Data points are jittered horizontally for visual clarity. Shallow+red spectrum at 350 µmol photons m^{-2} s^{-1} only.

Table S1. Linear model test results of relationship between chromophore / phycobilin concentrations and factors light intensity and spectral composition. Significant results highlighted in bold. PEB = phycoerythrobilin, PUB = phycourobilin, PCB = phycocyanobilin, Chl = chlorophyll-a, PE = phycoerythrin, PC = phycocyanin, APC = allophycocyanin, PBS : Chl = ratio between total phycobilisome (PE+PC+APC+PUB) and Chl; M = mesophotic spectrum; S = shallow spectrum; R = shallow+red spectrum. Data transformations, where required to meet test assumptions, are indicated by [*x*]. N = 4 aquaria per treatment. SS = sum of squares; df = degrees of freedom.

	**Anova**				**Posthoc test results – between levels of Spectrum**				**Posthoc test results – between levels of Intensity (µmol photons m^{-2} s^{-1})**			
	SS	*df*	*F*	*p*	*Contrast*	*df*	*t ratio*	*p*	*Contrast*	*df*	*t ratio*	*p*
	**PEB [log_{10}]**											
Intensity	0.11129	3	1.084	0.373	Averaged over Intensity							
Spectrum	0.6076	2	8.877	**0.001**	D – S	27	-1.665	0.237				
Intensity *					Within 350 µmol photons m^{-2} s^{-1} intensity							
Spectrum	0.27187	3	2.648	0.069								
Residuals	0.92399	27			D – R	27	-4.953	**<0.001**				
					D – S	27	-3.202	**0.009**				
					R – S	27	1.751	0.205				
	**PUB (log_{10})**											
Intensity	0.121	3	1.379	0.270	Averaged over Intensity							
Spectrum	0.490	2	8.350	**0.002**	D – S	27	-1.590	0.267				
Intensity *	0.255	3	2.898	0.053	Within 350 µmol photons m^{-2} s^{-1} intensity							
Spectrum												
Residuals	0.792	27			D – R	27	-4.915	**<0.001**				
					D – S	27	-3.310	**0.007**				
					R – S	27	1.605	0.261				
	**PCB [1/x]**											
Intensity	0.0002	3	0.255	0.857	Averaged over Intensity							
Spectrum	0.002	2	3.760	**0.036**	D – S	27	0.625	0.808				

Intensity *					Within 350 µmol photons m^{-2} s^{-1} intensity							
Spectrum	0.001	3	2.162	0.116								
Residuals	0.006	27			D – R	27	3.562	**0.004**				
					D – S	27	2.499	**0.048**				
					R – S	27	-1.063	0.545				
	**Chl**											
Intensity	1146538	3	4.369	**0.012**					Averaged over levels of Spectrum:			
Spectrum	15060	2	0.086	0.918					2.5 – 20	27	-0.748	0.877
Intensity *									2.5 – 350	27		
Spectrum	318703	3	1.214	0.323							-2.688	**0.056**
Residuals	2361805	27							2.5 – 80	27	0.754	0.874
									20 – 350	27	-1.94	0.236
									20 – 80	27	1.501	0.451
									350 - 80	27	3.442	**0.010**
	**PE [cube root]**											
Intensity	0.227	3	0.922	0.444	Averaged over Intensity							
Spectrum	1.407	2	8.587	**0.001**	D – S	27	-1.596	0.265				
Intensity *	0.534	3	2.172	0.114	Within 350 µmol photons m^{-2} s^{-1} intensity							
Spectrum												
Residuals	2.212	27			D – R	27	-4.775	**<0.001**				
					D – S	27	-2.925	**0.018**				
					R – S	27	-1.850	0.173				
	**PC [1/x]**											
Intensity	2.4e-7	3	0.255	0.857	Averaged over Intensity							
Spectrum	2.4e-6	2	3.760	**0.036**	D – S	27	0.625	0.808				
Intensity *	2.0e-6				Within 350 µmol photons m^{-2} s^{-1} intensity							
Spectrum		3	2.162	0.116								
Residuals	8.4e-6	27			D – R	27	3.562	**0.004**				

					D – S	27	2.499	**0.047**				
					R – S	27	1.063	0.545				
	**APC [log$_{10}$]**											
Intensity	0.117	3	0.330	0.804								
Spectrum	0.244	2	1.035	0.369								
Intensity * Spectrum	0.571	3	1.615	0.209								
Residuals	3.184	27										
	**PBS : Chl [log$_{10}$]**											
Intensity	0.374	3	4.494	**0.011**	Intensity = 2.5 µmol photons m^{-2} s^{-1}				Spectrum = mesophotic. Averaged over levels of Months			
Spectrum	0.606	2	10.922	**<0.001**	M – S	27	1.625	0.253	2.5 – 20	27	1.486	0.459
Intensity * Spectrum	0.539	3	6.478	**0.002**	Intensity = 20 µmol photons m^{-2} s^{-1}				2.5 – 350	27		
											1.045	0.725
Residuals	0.749	27			M – S	27	-0.802	0.705	2.5 – 80	27	0.989	0.757
					Intensity = 80 µmol photons m^{-2} s^{-1}				20 – 350	27	-0.441	0.971
					M – S	27	-0.173	0.984	20 – 80	27	-0.497	0.959
					Intensity = 350 µmol photons m^{-2} s^{-1}				350 - 80	27	0.056	1.000
					M – S	27	-5.920	**<0.001**	Spectrum = shallow. Averaged over levels of Months			
					M – R	27	2.499	**0.048**	2.5 – 20	27	-0.941	0.783
					S – R	27	1.481	0.316	2.5 – 350	27	-5.019	**<0.001**
									2.5 – 80	27	-0.81	0.849
									20 – 350	27	-4.079	**0.002**
									20 – 80	27	0.131	0.999
									350 - 80	27	-4.21	**0.001**

Table S2. Linear model test results of relationship between PE:PC concentration and EET ratios and chromophore EET with factors light intensity and spectral composition. Significant results highlighted in bold. PE = phycoerythrin; PC = phycocyanin; EET = excitation energy transfer, PEB = phycoerythrobilin, PUB = phycourobilin, PCB = phycocyanobilin; M = mesophotic spectrum; S = shallow spectrum; R = shallow + red spectrum. For PE:PC concentration, N = 4 per treatment. For all EETs, N = 3-4 per treatment. SS = sum of squares; df = degrees of freedom.

	**Anova**				**Posthoc test results – between levels of Spectrum**				**Posthoc test results – between levels of Intensity (μmol photons m^{-2} s^{-1})**			
	SS	*df*	*F*	*p*	*Contrast*	*df*	*t ratio*	*p*	*Contrast*	*df*	*t ratio*	*p*
	**PE:PC concentration**											
Intensity	12.899	3	2.2704	0.103	Averaged over Intensity							
Spectrum	13.442	2	3.5491	**0.042**	D – S	27	-0.701	0.765				
Intensity *					Within 350 μmol photons m^{-2} s^{-1} intensity							
Spectrum	14.312	3	2.5192	0.079								
Residuals	51.13	27			D – R	27	2.635	**0.036**				
					D – S	27	0.818	0.695				
					R – S	27	1.817	0.183				
	**PEB:PCB EET**											
Intensity	0.928	3	1.971	0.146								
Spectrum	0.574	2	1.828	0.183								
Intensity *												
Spectrum	1.063	3	2.258	0.109								
Residuals	3.611	23										
	**PEB EET (log_{10})**											
Intensity					Intensity = 2.5 μmol photons m^{-2} s^{-1}				Spectrum = mesophotic. Averaged over levels of Months			
	0.001	3	2.347	0.103								
Spectrum	0.002	2	4.871	0.019	M – S	20	-4.408	**<0.001**	2.5 – 20	20	-3.412	**0.014**

Intensity *					Intensity = 20 µmol photons m^{-2} s^{-1}				2.5 – 350	20	-2.997	**0.033**
Spectrum	0.005	3	8.379	**0.001**								
Residuals	0.004	20			M – S	20	1.311	0.406	2.5 – 80	20	0.705	0.894
					Intensity = 80 µmol photons m^{-2} s^{-1}				20 – 350	20	0.055	1.000
					M – S	20	-3.616	**0.005**	20 – 80	20	4.117	**0.003**
					Intensity = 350 µmol photons m^{-2} s^{-1}				350 - 80	20	3.627	**0.008**
					M – S	20	0.692	0.770	Spectrum = shallow. Averaged over levels of			
									Months			
					M – R	20	0.761	0.731	2.5 – 20	20	2.377	0.114
					S – R	20	0.035	0.999	2.5 – 350	20	1.832	0.288
									2.5 – 80	20	1.850	0.280
									20 – 350	20	-0.418	0.975
									20 – 80	20	-0.569	0.940
									350 - 80	20	-0.108	1.000
	**PUB EET (sqrt)**											
Intensity					Intensity = 2.5 µmol photons m^{-2} s^{-1}				Spectrum = mesophotic. Averaged over levels			
	0.201	3	1.088	0.377					of Months			
Spectrum	0.368	2	2.987	0.073	M – S	20	3.128	**0.014**	2.5 – 20	20	3.15	**0.024**
Intensity *					Intensity = 20 µmol photons m^{-2} s^{-1}				2.5 – 350	20		
Spectrum	1.090	3	5.902	**0.005**							2.179	0.163
Residuals	1.231	20			M – S	20	-1.826	0.187	2.5 – 80	20	-0.269	0.993
					Intensity = 80 µmol photons m^{-2} s^{-1}				20 – 350	20	-0.638	0.919
					M – S	20	3.228	**0.011**	20 – 80	20	-3.419	**0.013**
					Intensity = 350 µmol photons m^{-2} s^{-1}				350 - 80	20	-2.42	0.105
					M – S	20	0.340	0.939	Spectrum = shallow. Averaged over levels of			
									Months			
					M – R	20	0.343	0.938	2.5 – 20	20	-1.803	0.301
					S – R	20	-0.017	1.000	2.5 – 350	20	-0.312	0.989

									2.5 – 80	20	-0.404	0.977
									20 – 350	20	1.47	0.473
									20 – 80	20	1.511	0.450
									350 - 80	20	-0.071	1.000
	**PCB EET**											
Intensity					Intensity = 2.5 µmol photons m^{-2} s^{-1}				Spectrum = mesophotic. Averaged over levels			
	9.314	3	1.119	0.365					of Months			
Spectrum	8.362	2	1.507	0.246	M – S	20	-1.500	0.312	2.5 – 20	20	-2.536	0.084
Intensity *					Intensity = 20 µmol photons m^{-2} s^{-1}				2.5 – 350	20		
Spectrum	33.370	3	4.009	**0.022**							-1.226	0.618
Residuals	55.497	20			M – S	20	2.013	0.135	2.5 – 80	20	-0.1	1.000
					Intensity = 80 µmol photons m^{-2} s^{-1}				20 – 350	20	1.043	0.727
					M – S	20	-2.604	**0.043**	20 – 80	20	2.436	0.102
					Intensity = 350 µmol photons m^{-2} s^{-1}				350 - 80	20	1.136	0.672
					M – S	20	-1.219	0.456	Spectrum = shallow. Averaged over levels of			
									Months			
					M – R	20	-1.303	0.410	2.5 – 20	20	0.905	0.802
					S – R	20	-0.020	1.000	2.5 – 350	20	-1.233	0.614
									2.5 – 80	20	-1.107	0.689
									20 – 350	20	-2.223	0.151
									20 – 80	20	-2.174	0.165
									350 - 80	20	0.211	0.997
	**PUB : PEB EET**											
Intensity					Intensity = 2.5 µmol photons m^{-2} s^{-1}				Spectrum = mesophotic. Averaged over levels			
	0.022	3	0.850	0.481					of Months			
Spectrum	0.066	2	3.768	**0.039**	M – S	20	-1.500	0.312	2.5 – 20	20	-2.536	0.084
Intensity *					Intensity = 20 µmol photons m^{-2} s^{-1}				2.5 – 350	20		
Spectrum	0.195	3	7.426	**0.001**							-1.226	0.618

Residuals	0.193	22			M – S	20	2.013	0.135	2.5 – 80	20	-0.1	1.000
					Intensity = 80 µmol photons m^{-2} s^{-1}				20 – 350	20	1.043	0.727
					M – S	20	-2.604	**0.043**	20 – 80	20	2.436	0.102
					Intensity = 350 µmol photons m^{-2} s^{-1}				350 - 80	20	1.136	0.672
					M – S	20	-1.219	0.456	Spectrum = shallow. Averaged over levels of Months			
					M – R	20	-1.303	0.410	2.5 – 20	20	0.905	0.802
					S – R	20	-0.020	1.000	2.5 – 350	20	-1.233	0.614
									2.5 – 80	20	-1.107	0.689
									20 – 350	20	-2.223	0.151
									20 – 80	20	-2.174	0.165
									350 - 80	20	0.211	0.997

Table S3. Linear mixed model test results of relationship between absorbance derived chromophore / phycobilin ratios of *L. glaciale* and predictor variables light intensity (categorical), spectral composition (categorical) and time (categorical), with "aquarium" included as a random variable. *P*-values for posthoc comparisons use Kenward-Roger approximation for the degrees of freedom (df) with Tukey adjustment. Significant results (at $p<0.05$) highlighted in bold. PEB = phycoerythrobilin, PUB = phycourobilin, PCB = phycocyanobilin, M = mesophotic spectrum; S = shallow spectrum; R = shallow + red spectrum. Comparison level is indicated in [x]. N = 4 aquaria per treatment; observations = 108. SS = sum of squares; df = degrees of freedom.

	**Anova (type III)**				**Posthoc test results – between levels of Spectrum**				**Posthoc test results – between levels of Intensity (µmol photons m^{-2} s^{-1})**			
	SS	*df*	*F*	*p*	*Contrast*	*df*	*t ratio*	*p*	*Contrast*	*df*	*t ratio*	*p*
	**PEB:PCB absorbance**											
Intensity					Intensity = 2.5 µmol photons m^{-2} s^{-1}				Spectrum = mesophotic. Averaged over levels of Months			
	0.3362	3	10.7233	**<0.001**								
Spectrum	0.0178	2	0.8526	0.438	M – S	27	-0.427	0.905	2.5 – 20	27	1.506	0.448
Months	7.4992	2	358.8316	**<0.001**	Intensity = 20 µmol photons m^{-2} s^{-1}				2.5 – 350	27	1.396	0.513
Intensity *					M – S	27	0.537	0.854	2.5 – 80	27		
Spectrum	0.0942	3	3.0042	**0.048**							4.156	**0.002**
					Intensity = 80 µmol photons m^{-2} s^{-1}				20 – 350	27	-0.11	1.000
					M – S	27	0.625	0.808	20 – 80	27	2.651	0.060
					Intensity = 350 µmol photons m^{-2} s^{-1}				350 - 80	27	2.76	**0.048**
					M – S	27	-3.088	**0.012**	Spectrum = shallow. Averaged over levels of Months			
					M – R	27	-2.034	0.123	2.5 – 20	27	0.542	0.948
					S – R	27	-1.054	0.550	2.5 – 350	27	4.057	**0.002**
									2.5 – 80	27	3.104	**0.022**
									20 – 350	27	3.515	**0.008**
									20 – 80	27	2.562	0.073
	**PUB : (PEB+PUB+PCB) absorbance**											
Intensity	0.000233	3	2.0937	0.124	Averaged over Intensity							

Spectrum	0.000361	2	4.8689	**0.015**	D – S	27	-3.057	**0.013**				
Months	0.188282	2	2538.949	<**0.001**	Within 350 µmol photons m^{-2} s^{-1} intensity							
Intensity *					D – R	27	-3.768	**0.002**				
Spectrum	0.000295	3	2.6539	0.069								
					D – S	27	-2.428	**0.056**				
					R – S	27	-1.341	0.386				
	**PEB: (PEB+PUB+PCB) absorbance**											
Intensity									Averaged over levels of Spectrum (D & S),			
	0.000349	3	19.6982	<**0.001**					Months:			
Spectrum	2.71E-05	2	1.5294	0.235					2.5 – 20	27	0.423	0.974
Months	0.009886	2	557.5081	<**0.001**					2.5 – 350	27	6.016	<**0.001**
Intensity *									2.5 – 80			
Spectrum	3.28E-05	3	1.852	0.162						27	5.2	<**0.001**
									20 – 350	27	5.593	<**0.001**
									20 – 80	27	4.777	<**0.001**
									350 - 80	27	-0.816	0.846
	**PC: (PEB+PUB+PCB) absorbance**											
Intensity									Averaged over levels of Spectrum (D & S),			
	0.000773	3	8.2459	<**0.001**					Months:			
Spectrum	0.000079	2	1.2607	0.300					2.5 – 20	27	-1.375	0.525
Months	0.088257	2	1412.614	<**0.001**					2.5 – 350	27	-3.393	**0.011**
Intensity *									2.5 – 80			
Spectrum	0.000248	3	2.65	0.069						27	-4.543	**0.001**
									20 – 350	27	-2.017	0.207
									20 – 80	27	-3.168	**0.019**
									350 - 80	27	-1.151	0.662

Table S3 continued

Posthoc test results – between levels of Months. Averaged over levels of Spectrum and Intensity.

Contrast	*df*	*t ratio*	*p*
**PEB:PCB absorbance**			
3 – 6	70	-19.846	**<0.001**
3 - 9	70	5.661	**<0.001**
6 - 9	70	25.507	**<0.001**
**PUB : (PEB+PUB+PCB) absorbance**			
3 – 6	70	-58.369	**<0.001**
3 - 9	70	6.217	**<0.001**
6 - 9	70	64.586	**<0.001**
**PEB: (PEB+PUB+PCB) absorbance**			
3 – 6	70	30.497	**<0.001**
3 - 9	70	3.472	**0.003**
6 - 9	70	- 27.026	**<0.001**
**PC: (PEB+PUB+PCB) absorbance**			
3 – 6	70	40.613	**<0.001**
3 - 9	70	-9.389	**<0.001**
6 - 9	70	-50.002	**<0.001**

Table S4. Linear mixed model test results of relationship between photosynthetic characteristics of *L. glaciale* and predictor variables light intensity (categorical), spectral composition (categorical) and time (categorical), with "aquarium" included as a random variable. *P*-values for posthoc comparisons use Kenward-Roger approximation for the degrees of freedom (df) with Tukey adjustment. Significant results (at $p<0.05$) highlighted in bold. Comparison level is indicated in [x]. N = 4 aquaria per treatment; observations = 108. M = mesophotic spectrum; S = shallow spectrum; R = shallow + red spectrum.

	**Anova (type III)**				**Posthoc test results – between levels of Spectrum**				**Posthoc test results – between levels of Intensity (μmol photons m^{-2} s^{-1})**			
	SS	*df*	*F*	*p*	*Contrast*	*df*	*t ratio*	*p*	*Contrast*	*df*	*t ratio*	*p*
	$\boldsymbol{E_k}$											
Intensity									Averaged over levels of Spectrum (D & S),			
	75.833	3	57.5884	**<0.001**					Months:			
Spectrum	1.038	2	1.1826	0.311					2.5 – 20	27	-1.258	0.597
Months	48.009	2	54.6883	**<0.001**					2.5 – 350	27	-11.923	**<0.001**
Intensity *									2.5 – 80			
Spectrum	1.204	3	0.9143	0.437						27	-5.25	**<0.001**
									20 – 350	27	-10.665	**<0.001**
									20 – 80	27	-3.993	**0.002**
									350 - 80	27	6.673	**<0.001**
	**ETRmax**											
Intensity					Intensity = 2.5 μmol photons m^{-2} s^{-1}				Spectrum = mesophotic. Averaged over levels			
	0.21276	3	2.5287	0.062					of Months			
Spectrum	0.29349	2	5.2324	**0.007**	M – S	27	-0.267	0.962	2.5 – 20	27	-1.248	0.603
Months	1.83831	2	32.7736	**<0.001**	Intensity = 20 μmol photons m^{-2} s^{-1}				2.5 – 350	27	-0.884	0.813
Intensity *					M – S	27	-0.321	0.945	2.5 – 80	27		
Spectrum	0.25372	3	3.0156	**0.034**							0.526	0.952
					Intensity = 80 μmol photons m^{-2} s^{-1}				20 – 350	27	0.364	0.983
					M – S	27	3.267	**0.008**	20 – 80	27	1.773	0.308
					Intensity = 350 μmol photons m^{-2} s^{-1}				350 - 80	27	1.409	0.505

					M – S	27	1.780	0.195	Spectrum = shallow. Averaged over levels of Months			
					M – R	27	2.920	**0.019**	2.5 – 20	27	-1.194	0.636
					S – R	27	-1.139	0.499	2.5 – 350	27	-2.931	**0.032**
									2.5 – 80	27	-3.009	**0.027**
									20 – 350	27	-1.737	0.325
									20 – 80	27	-1.815	0.289
									350 - 80	27	-0.078	1.000
	**log(a)**											
Intensity	0.130195	3	30.093	**<0.001**					Averaged over levels of Spectrum (D & S), Months:			
Spectrum	0.006718	2	2.3292	0.103					2.5 – 20	27	-5.977	**<0.001**
Months	0.041084	2	14.2443	**<0.001**					2.5 – 350	27	-9.163	**<0.001**
Intensity * Spectrum	0.011093	3	2.5641	0.059					2.5 – 80	27	-6.622	**<0.001**
									20 – 350	27	-3.186	**0.018**
									20 – 80	27	-0.646	0.916
									350 - 80	27	2.54	0.076
	**log(F_v/F_m)**											
Intensity	0.130305	3	30.1177	**<0.001**					Averaged over levels of Spectrum (D & S), Months:			
Spectrum	0.006805	2	2.3594	0.100					2.5 – 20	27	-5.979	**<0.001**
Months	0.040776	2	14.1368	**<0.001**					2.5 – 350	27	-9.165	**<0.001**
Intensity * Spectrum	0.011058	3	2.5559	0.060					2.5 – 80	27	-6.63	**<0.001**
									20 – 350	27	-3.186	0.018
									20 – 80	27	-0.651	0.914
									350 - 80	27	2.535	0.077

Table S4 continued.

Posthoc test results – between levels of Months. Averaged over levels of Spectrum and Intensity.

Contrast	*df*	*t ratio*	*p*
**Ek**			
3 – 6	70	3.494	**0.002**
3 - 9	70	10.284	**<0.001**
6 - 9	70	6.790	**<0.001**
**ETRmax**			
3 – 6	70	5.423	**<0.001**
3 - 9	70	7.918	**<0.001**
6 - 9	70	2.495	**0.039**
**log(a)**			
3 – 6	70	-2.484	**0.040**
3 - 9	70	-5.333	**<0.001**
6 - 9	70	-2.850	**0.016**
**log(Fv/Fm)**			
3 – 6	70	-2.470	**0.042**
3 - 9	70	-5.313	**<0.001**
6 - 9	70	- 2.843	**0.016**
